# Screening for an ivermectin slow-release formulation suitable for malaria vector control

**DOI:** 10.1186/s12936-015-0618-2

**Published:** 2015-03-05

**Authors:** Carlos Chaccour, Ángel Irigoyen Barrio, Ana Gloria Gil Royo, Diego Martinez Urbistondo, Hannah Slater, Felix Hammann, Jose Luis Del Pozo

**Affiliations:** Department of Internal Medicine, Clinica Universidad de Navarra, Pio XII 36, Pamplona, 31008 Spain; Barcelona Institute for Global Health (ISGlobal), Barcelona, Spain; Instituto de Salud Tropical, Universidad de Navarra, Pamplona, Spain; Faculty of Pharmacy, Universidad de Navarra, Pamplona, 31008 Spain; Drug Development Unit Universidad de Navarra (DDUNAV), Pamplona, Spain; Department of Infectious Disease Epidemiology, MRC Centre for Outbreak Analysis and Modelling, Imperial College London, London, UK; Cantonal Hospital Baselland, Medical University Clinic, Liestal, Switzerland; Infectious Disease Unit, Clinica Universidad de Navarra, Pamplona, Spain; Department of Microbiology, Clinica Universidad de Navarra, Pamplona, Spain

**Keywords:** Vector control, Malaria, Malaria elimination, Ivermectin, Slow release, *Anopheles gambiae*, Silicone, Residual transmission, Outdoor transmission

## Abstract

**Background:**

The prospect of eliminating malaria is challenged by emerging insecticide resistance and vectors with outdoor and/or crepuscular activity. Ivermectin can simultaneously tackle these issues by killing mosquitoes feeding on treated animals and humans. A single oral dose, however, confers only short-lived mosquitocidal plasma levels.

**Methods:**

Three different slow-release formulations of ivermectin were screened for their capacity to sustain mosquito-killing levels of ivermectin for months. Thirty rabbits received a dose of one, two or three silicone implants containing different proportions of ivermectin, deoxycholate and sucrose. Animals were checked for toxicity and ivermectin was quantified periodically in blood. Potential impact of corresponding long-lasting formulation was mathematically modelled.

**Results:**

All combinations of formulation and dose released ivermectin for more than 12 weeks; four combinations sustained plasma levels capable of killing 50% of *Anopheles gambiae* feeding on a treated subject for up to 24 weeks. No major adverse effects attributable to the drug were found. Modelling predicts a 98% reduction in infectious vector density by using an ivermectin formulation with a 12-week duration.

**Conclusions:**

These results indicate that relatively stable mosquitocidal plasma levels of ivermectin can be safely sustained in rabbits for up to six months using a silicone-based subcutaneous formulation. Modifying the formulation of ivermectin promises to be a suitable strategy for malaria vector control.

**Electronic supplementary material:**

The online version of this article (doi:10.1186/s12936-015-0618-2) contains supplementary material, which is available to authorized users.

## Background

Increased funding and political commitment have led to outstanding worldwide achievements in malaria control over the last 14 years. The estimated malaria mortality rate in children under five has almost been halved worldwide since 2000 and it is projected to decrease by 61% by 2015 [1]. Yet, malaria is still a formidable public health problem that caused an estimated 198 million cases and 584,000 deaths in 2013 [1]. In many settings, there has been a renewed interest for malaria elimination [2], and the general mood is that of ‘impatient optimism’ [3]. This general optimism has translated into more concrete research [4] and operational [5] agendas to decrease malaria transmission until eradication. The main determinants of malaria transmission are mosquito-related variables [6]; vector control plays a central role in control strategies [7]. It has been the most successful intervention in the past [8] and it is likely to remain so in future elimination endeavours.

The malERA consultative group on vector control identified three main challenges to eradication [9]: 1) the emergence of insecticide resistance affecting all major vector species and all classes of insecticides in two-thirds of endemic countries [7]; 2) the presence of outdoor-biting/resting mosquitoes, not readily targeted by insecticide-treated nets (ITNs) and indoor-residual spraying (IRS) [10,11]; and, 3) the need for new approaches to achieve elimination in areas where vectors exhibit a particularly high vectorial capacity. Additional problems include the selection of vectors with early or crepuscular activity in areas with good ITN coverage [12,13], vector biodiversity and environmental change [14].

One additional source of concern for eradication is the emergence of artemisinin resistance in Southeast Asia. The historic perspective is unsettling as this area has repeatedly been the epicentre from where resistance to anti-malarial drugs has spread to Africa and the rest of the world [15]. An emergency containment plan is in place [16] and even focal elimination of all falciparum malaria has been advocated [17,18]. One further problem is that the main local malaria vectors, *Anopheles dirus* and *Anopheles minimus*, show substantial outdoor feeding and biting [19] as well as crepuscular activity and a tendency to bite early at night [20], which limits the effectiveness of ITNs and IRS. Additional vector control methods are urgently needed in the region [19].

Ivermectin (IVM) is a systemic insecticide that reduces the survival of mosquitoes feeding on treated humans, both under laboratory [21,22] and field conditions [23], potentially leading to a disruption in malaria transmission [24]. Mass drug administration (MDA) with IVM has been advocated as a complementary vector control strategy [25,26]. Potential benefits include: i) a novel mechanism of action [27] compared to currently used insecticides, which could circumvent resistance; ii) effectiveness against malaria vectors regardless of place or time of the feeding; and, iii) additional effects inhibiting *Plasmodium* sporogony [28].

All these characteristics make MDA with IVM a particularly valuable intervention for the vectors in the Mekong region and local elimination endeavours in the light of artemisinin resistance. According to a recent mathematical model [29], a key factor for interrupting transmission would be the time IVM remains in blood above mosquito-killing levels.

The lethal concentration 50 (LC_50_) is defined as the blood levels needed in order to kill 50% of the mosquitoes feeding on a treated individual, its closest clinical equivalent is the minimum inhibitory concentration used in microbiology labs. The LC_50_ of IVM for *Anopheles gambiae* in the first ten days after a blood meal has been estimated by mixing human blood with a known dilution of the drug in a tube and then performing a membrane feeding essay. The LC_50_ estimated with this method ranges from 14.6 to 26.9 ng/ml [22,28]. Recent evidence from membrane feedings using blood drawn from ivermectin treated volunteers gives a much lower range of 4.69-7.51 ng/ml [30]. This discrepancy could be the result of unknown active metabolites and possibly, blood meal size differences between membrane and skin feeding mosquitoes.

At the approved dose of 200 μg/kg, current oral formulations can only maintain mosquitocidal concentrations for approximately 48 hours [31,32]. Alternatives include a scheme with multiple doses over the course of weeks, which poses logistical challenges, or the administration of a slow-release formulation on one single encounter. Given IVM’s pharmacokinetic properties [33], a slow-release oral tablet could only increase the time with concentrations above LC_50_ in hours, while injectable, depot formulations could do it for days to weeks. An implantable subcutaneous device [34,35] could sustain key mosquito-killing levels of IVM for months. Subcutaneous implants for contraceptive purposes were licensed more than 30 years ago; they release small amounts of hormones for years and have an excellent security profile [36]. In developing countries suitable for the implementation of slow-release IVM formulations, the acceptance of contraceptive implants is high [37] and seems to be increasing [38].

The main goal of this work was to adapt the design of an IVM slow-release formulation to make it suitable for use in humans and peridomestic animals as an additional malaria vector control intervention. For this, three different IVM-containing silicone implant formulations were screened at different doses in a proof-of-concept animal model. Rabbits were chosen because their weight allows for an easier dose extrapolation to larger mammals.

## Methods

### The implants

**Figure 1:**
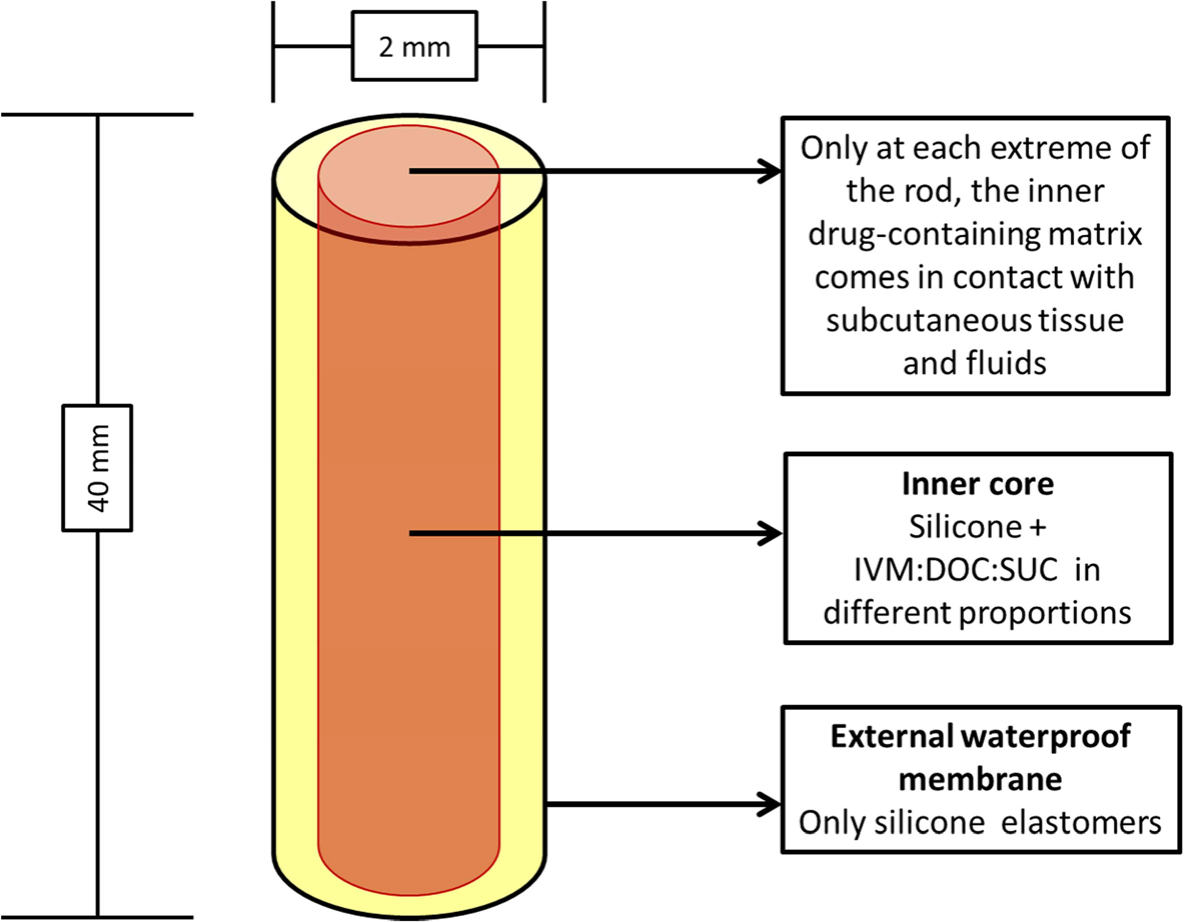
**Implant’s design and measures.** With the current measures, the elution surface of each implant is 6.28 sq mm (2 x π x 1^2^). IVM: ivermectin; DOC: deoxycholate; SUC: sucrose.

The 40×2-mm implants consist of two concentric cylinders (silicone-covered rod formulation). The external cylinder is a 100% silicone impermeable membrane; the inner cylinder contains silicone and a mixture of IVM, deoxycholate sodium (DOC) and sucrose (SUC). The inner drug-containing matrix contacts the subcutaneous tissue and fluids only at the ends, where the cylinders are cross-sectioned (Figure 1). Water enters at each end, dissolves the IVM-DOC-SUC mixture and creates microchannels in the inner core, allowing for a controlled release of the drug.

IVM is a lipophilic drug. DOC and SUC modify the solubility of IVM and change its release rate from the microchannels in the core. Two previously used proportions of IVM:DOC:SUC known to have an appropriate release profile in mice [35] and dogs [34] were chosen. An third formulation with a high proportion of DOC, which markedly increases the release rate, was also included. This resulted in a different total IVM content for each formulation. The internal cylinder (1.8 × 40 mm) in the implants allows for an elution surface of 5.08 sq mm each (2 π r^2^). This surface increases arithmetically when using higher doses (two or three implants). The total IVM calculated content of each formulation can be seen in Table 1. Implants were manufactured by Specialty Silicone Fabricators (SSFAb) at their ISO 13485-certified and FDA-registered facility in Tustin, California. The manufacture started my mixing the silicone component with the drug and excipients (DOC and SUC) and molding it in cylindrical shape to form the inner drug matrix core. The outer high consistency rubber layer was extruded separately and applied over the drug core to complete the implants. See references [34,35] for a detailed description of the production methods. The rods were sterilized using an electron beam.

**Table 1:** **Composition and total ivermectin content of the implants according to formulation**

**Formulation**	**IVM**	**DOC**	**SUC**	**Total IVM/rod**
F	80%	13%	7%	29 mg
M	50%	33%	17%	18 mg
X	35%	55%	10%	13 mg

Calculations done using the volume of the inner rod (0.102 cc) and assuming negligible changes in the density of silicone (1.16 g/cc) by adding the IM-DOC-SUC powder.

IVM: ivermectin; DOC: deoxycholate; SUC: sucrose.

### Procedures

See Table 2 for a timeline of all procedures. The subjects were 30 male New Zealand white rabbits of 16 weeks of age. The diet was limited to 250 g/day in an attempt to keep the weight stable. The animals were kept in individual cages. Appropriate environmental enrichment was used. Rabbits were randomly marked in the ear with numbers 1–30 by the provider. Animals were assigned to each formulation (F, M, X) in numerical order. In each formulation group, the rabbits were assigned to a subgroup receiving a dose of one, two or three rods (1F, 2F, 3F, 1M, 2M, 3M, 1X, 2X, 3X). Each subgroup contained three animals (in total 27 intervention plus three controls). Control animals received one, two or three 100% silicone rods. The rods were inserted subcutaneously between the scapulae by means of a Jadelle® trocar under general anaesthesia. When more than one implant was inserted, they were placed forming a ‘V’ shape.

**Table 2:** **Timeline of procedures**

**Intervention**	**Weeks**																									
	**0**	**1**	**2**	**3**	**4**	**5**	**6**	**7**	**8**	**9**	**10**	**11**	**12**	**13**	**14**	**15**	**16**	**17**	**18**	**19**	**20**	**21**	**22**	**23**	**24**	**25**
**Implantation**		●																								
**Check up**	●	●	●	●	●	●	●	●	●	●	●	●	●	●	●	●	●	●	●	●	●	●	●	●	●	●
**Vital signs**	●	●											●												●	
**ECG**	●	●											●												●	
**Ophthalmoscopy**	●												●												●	
**Toxicology samples**	●	●											●												●	
**IVM PK samples**	●	●	●	●	●		●	●	●	●	●	●	●				●				●				●	
**Interim analysis**														●												
**Euthanasia (1/2F, 1/2M, 1X)**															●											
**Euthanasia (3F, 3M, 2/3X)**																										●

IVM: ivermectin; PK: pharmacokinetics.

After implantation, the animals were checked daily for clinical signs of IVM toxicity such as sleepiness, ataxia and increase in tone [39]. Blood for IVM quantification was drawn weekly for the first 12 weeks, under sedation, from the marginal ear vein. An interim analysis of plasma levels was performed at 13 weeks and only groups where all subjects maintained at least 10 ng/ml for the whole period were continued until week 25. Blood samples were taken monthly in the remaining groups until euthanasia. IVM plasma levels were determined using a variation of a previously described HPLC-FLD [40,41]. The method was validated in accordance with the European Medicines Agency guidelines [42].

A comprehensive toxicological profile was performed, reviewing the neurological, cardiovascular, respiratory, haematologic, digestive, and urinary systems. For toxicological analysis, additional blood was drawn at baseline and at 12 and 24 weeks. Blood tests included full count, coagulation panel, glucose, electrolytes, muscular and liver enzymes, bilirubin, cholesterol, total proteins, and albumin. A urinary dipstick test was performed before euthanasia. Vital signs were assessed at baseline and in weeks 1, 12 and 24 after implantation together with ECGs and indirect ophthalmoscopic examination. QT intervals in the ECG were corrected using Bazett’s formula. Normal values for vital signs, ECG, haematology, coagulation, and biochemistry were taken from the literature [43] and compared to the mean and 95% CI of the baseline values.

Euthanasia was performed under sedation using T61® (MSD) at 25 weeks. An experienced toxicologist performed full macroscopic autopsies on all rabbits. All procedures were reviewed and approved by the animal experimentation ethics committee of the Universidad de Navarra (Registry number CEEA/135-12).

### Pharmacokinetic calculations and statistics

Pharmacokinetic calculations were performed with Mathematica, Version 8.0, Wolfram Research, Inc., Champaign, IL, USA (2010). Areas under the curve (AUC) were calculated using the linear trapezoidal rule.

ANOVA and Chi square test were used to contrast the AUC of the different formulation-dose combinations at 12 weeks and the role of the elution area of the implants on the likelihood of maintaining IVM plasma level above 7 ng/ml (the estimated *in vivo* LC_50_) for the same period of time. Furthermore, a univariate linear regression model was performed to assess the capacity of the formula (proportion of DOC x elusion surface) to predict the AUC at 12 weeks.

Comparisons of vital signs and blood test results at baseline and at 1 and 12 weeks after implantation were done using T-test for paired samples. For comparing baseline results with those of 24 weeks, the Wilcoxon paired test was used. Statistical analysis was done with SPSS version 20.0.

### Mathematical modelling of potential impact

The mathematical model developed by Slater *et al*. [29] was used to assess the potential impact of a long-lasting IVM product on mosquito survival and malaria incidence in humans. The model considered a formulation capable of sustaining 8 ng/ml plasma levels for two, four, eight, 12 or 24 weeks administered to 80% of the population over five years of age. This concentration is calculated to increase the daily hazard of mortality to mosquitoes by 4.4 times, resulting in the mean lifespan of a mosquito in the wild decreasing from 7.6 days [44] to 1.7 days.

## Results

### Plasma levels and pharmacokinetics

**Figure 2:**
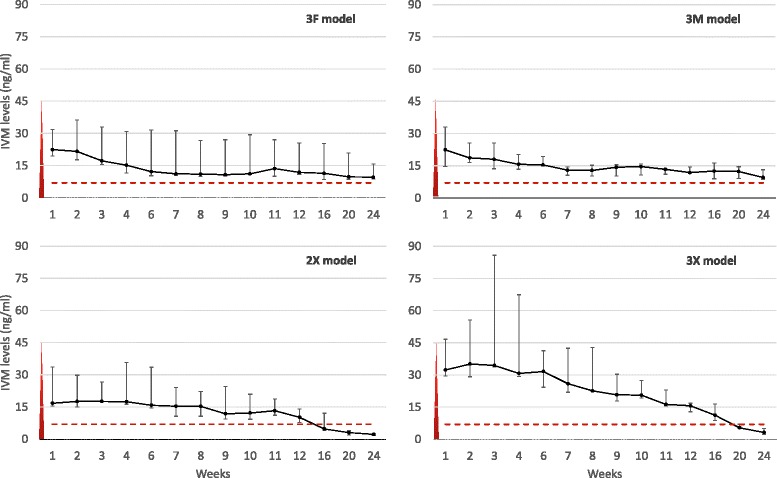
**Pharmacokinetic curves of the leading formulations.** PK curves (median and range) of the four groups that maintained IVM levels of at least 10 ng/ml for the first 12 weeks and were selected to continue until week 25. The red triangle is the approximate PK curve of one single 150 μg/kg oral dose. The dotted line marks 7 ng/ml, the minimum LC_50_ determined *in vivo* for *An. gambiae*.

All combinations of formulation and dose released IVM for more than 12 weeks. Table 3 shows the median and range of main pharmacokinetics (PK) parameters in the different groups. Figure 2 shows the PK curves of the four groups that maintained IVM levels of at least 10 ng/ml for the first 12 weeks. These were selected to continue until week 25 (3F, 3M, 2X, 3X).

**Table 3:** **Main pharmacokinetic parameters of all groups**

**Group**	**Mean daily dose**	**Tmax**	**Cmax**	**AUC 12 weeks**	**AUC 24 weeks**	**Time >7 ng/ml**
1 F	64 (60–77)	2 (1–2)	7 (7–10)	66 (61–68)	-	3 (1–3)
2 F	76 (75–105)	1 (1–2)	16 (12–16)	120 (113–163)	-	12 (11–12)
3 F	75 (70–85)	1 (1–2)	22 (19–36)	155 (153–348)	277 (266–615)	24 (24)
1 M	34 (31–43)	7 (7–11)	13 (4–20)	81 (38–126)	-	3 (0–11)
2 M	83 (77–91)	2 (1–3)	16 (14–20)	122 (106–164)	-	12 (8–12)
3 M	69 (63–73)	1 (1–2)	22 (16–33)	164 (160–224)	300 (278–403)	24 (24)
1X	30 (29–32)	3 (2–8)	12 (8–14)	79 (63–85)	-	2 (1–3)
2X	30 (27–34)	4 (3–4)	17 (17–35)	170 (152–311)	218 (207–404)	12 (12–16)
3X	43 (41–49)	3 (2–7)	42 (35–85)	307 (306–497)	422 (393–620)	16 (16)

For all values median and range are given. Mean daily dose (μg/kg/day), Tmax (weeks), Cmax (ng/ml), AUC (ng · week/ml), Time above 7 ng/ml (weeks).

**Figure 3:**
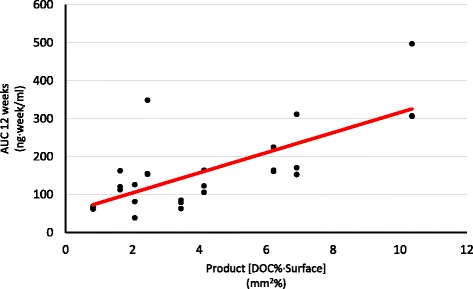
**Linear regression comparing area under the curve at 12 weeks with the product (DOC%•Surface).** T (magnitude) 5.34 (p < 0.01). Typified coefficient 0.73. DOC: deoxycholate.

Using ANOVA, a statistically significant difference in the AUC at 12 weeks between groups with different elution surface (dose) was found. Additionally, the Chi square test shows a significant difference in the likelihood of maintaining plasma levels above 7 ng/ml between the same groups. Neither of the tests demonstrates statistically significant differences in the same parameters when comparing groups with different formulations (DOC and SUC proportions) (Table 4). The linear regression model shows that the AUC at 12 weeks can be predicted using the product of the DOC proportion and the implant’s elusion surface (DOC% x surface). An increase of this product in 1 sq·mm% correlates with an increase in the AUC at 12 weeks of 26.45 ng · (week)/ml (p < 0.01) (Figure 3).

**Table 4:** **Statistic comparison of area under the curve and likelihood of maintaining plasma levels above 7 ng/ml for 12 weeks**

	**Magnitude**	**p**
**AUC at 12 weeks** ***vs*** **DOC% (ANOVA)**	F = 1.98	0.16
**AUC at 12 weeks** ***vs*** **surface (ANOVA)**	F = 12.37	<0.01
**12 weeks over 7 ng/ml** ***vs*** **DOC%**	Chi square = 0.37	0.86
**12 weeks over 7 ng/ml** ***vs*** **surface**	Chi square = 20.55	<0.01

Statistic comparison of AUC at week 12 and likelihood of maintaining plasma levels above 7 ng/ml for 12 weeks between groups with different dose and formulation.

### Toxicology

No major adverse effect attributable to the drug was found. See Additional files 1, 2 and 3 for the full toxicology report.

### Modelling

**Figure 4:**
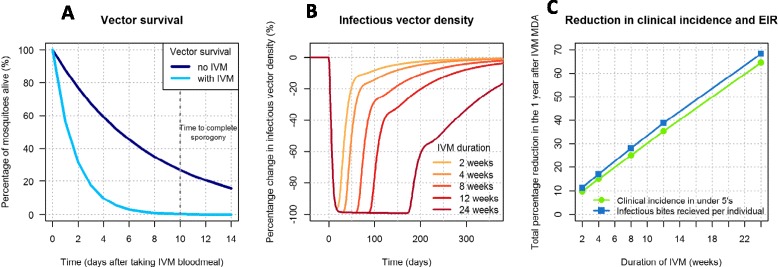
**Modelling the potential impact of slow-release ivermectin formulations.** Panel **A**: Expected change in vector survival in the presence of ivermectin treatment in the community. Panel **B**: Expected percentage change in the infectious vector density in time in the presence of different long-lasting ivermectin formulations. Panel **C**: Reductions in clinical incidence and entomological inoculation rate in the six months following implementation of a long-lasting ivermectin formulation.

Mosquito survival is assumed to be distributed exponentially; therefore, a population of vectors with a mean lifespan of 7.6 days will have a survival curve shown by the dark blue line in Figure 4A. Here, 27% of mosquitoes survive for the ten days required for the parasite to reach its infectious sporozoite stage. After an IVM blood meal containing 8 ng/ml, mean lifespan decreases to 1.7 days – this results in just 0.3% of mosquitoes surviving for ten days. Figure 4B shows how this reduced vector lifespan would impact the number of infectious vectors over time if IVM were given to 80% of the population over five years old. Figure 4C shows the cumulative reduction in clinical incidence in under fives and the number of infectious bites received per individual (the entomological inoculation rate (EIR)) in the six months following treatment. An IVM product with a two-week duration reduces the infectious vector population by over 90% for about four weeks, which results in a 20% reduction in clinical incidence in under fives and EIR. Infectious vector density can be almost totally suppressed (>98% reduction) for three months with an IVM product with a 12-week duration such as these implantables. A formulation with this duration of efficacy is estimated to reduce clinical incidence in under fives and EIR by >60% in the six months following implementation. Figure 4B and C show the expected proportional increase with formulations lasting for longer periods.

## Discussion

These results indicate that relatively stable plasma levels of IVM can be safely sustained in mammals for up to 24 weeks using a slow-release formulation. The insecticidal properties of IVM make it attractive as an alternative control tool for malaria [25] and other vector-borne diseases, including leishmaniasis and trypanosomiasis [27]. Its short half-life, however, could limit its widespread application. Modelling shows that the time it remains in plasma at levels lethal to vectors is critical for interrupting transmission [29].

Both the AUC and the time with plasma levels above 7 ng/ml (*An. gambiae*’s LC_50_ calculated *in vivo*) increase with the total elution surface of the formulations. This is a robust parameter likely to play an important role in the development of slow-release formulations due to its dose-dependent effect.

Formulations with a higher content of DOC tended to achieve markedly higher Cmax and AUC both at 12 and 24 weeks (though not statistically significant), possibly at the expense of time with a concentration above 7 ng/ml after 12 weeks, as seen in Figure 2. This is consistent with the increased IVM solubility caused by DOC [35], resulting in faster elution from the rods, i.e., ‘peaked’ PK curves. Formulations with less DOC content have release behaviour closer to zero order.

Using the formula (DOC% x elution surface) allows for a linear prediction of the AUC. Both factors (release area and excipients that increase solubility) seem to have this effect by increasing the Cmax, i.e., the ‘height’ of the curve. Another factor likely to have a linear influence on the AUC and the time above 7 ng/ml is the product (IVM% x elution surface x device volume). It was not possible to probe this theory because implants of equal volume were used. These factors, however, are likely to influence the AUC by increasing the time above target concentration, i.e., the ‘length’ of the curve. These concepts may assist in the design of different slow-release devices.

Given the drug’s lipophilic condition, an extrapolation of these results to humans was not ventured due to the disproportionate increase in adipose tissue when compared to rabbit. This might initially reduce the peak levels reached, but could effectively extend the time above target levels as the drug is release from adipose tissue.

No significant side effect could be attributed to the drug in this study. Although of minor clinical significance, future studies should assess a possible relationship with increased fibrinogen. It was not possible to find suitable data on proportional organ weight for laboratory rabbits of 40 weeks of age or older. The finding of a relative large spleen at 12 weeks post intervention should be interpreted carefully due to this lack of comparative standards and the absence of histological anomalies.

IVM is a safe drug. In ongoing MDA programmes for onchocerciasis and lymphatic filariasis, its benefits exceed the prevalence reduction of the target filariae as it has an impact on several soil-transmitted helminths and ectoparasites. A wider use of the drug as a public health tool has been advocated [45,46]. With appropriate resistance monitoring, assessment of pharmacokinetics and drug interactions issues, a slow-release IVM formulation could have additional value on the prevention and treatment of these [47] and other vector-borne diseases. There is a working delivery infrastructure for the drug used in MDA campaigns. The evaluation of these possibilities warrants joint work of the malaria and NTD communities.

### Limitations of this study

This study is intended as a proof of concept. As such it has several limitations. PK results are given as median and range due to the small sample size of each group. Additionally, findings in rabbits should be interpreted with caution due to possible differences in drug distribution and metabolism when compared with other mammals. Also, the rabbits gained an average 12% of their initial weight during the study, which could have affected total body fat and the drug’s PK. The manufacture of prototypes is not a standardized process and some PK variation may be caused by differences in implant weight affecting total drug content. The experiments described in this study were conducted under good laboratory practices (GLP), however, for budgetary reasons; the GLP certification was not sought. The modelling work is based on the hypothetical deployment of a new slow-release IVM product; it aims at elucidating the potential impact of such a long-lasting IVM formulation on malaria transmission. The data used to parameterize the model were based on mosquito mortality data taken from laboratory studies using IVM. The impact of a new formulation of IVM needs to be tested in the field to fully understand the impact on transmission. Potential challenges of implantable devices in the field include the need for a trained worker using a sterile technique for insertion and removal in case of adverse reactions, which might be local or systemic. Additionally, the silicone structure will remain in place after all the IVM and excipients have been released, the silicone itself is medical grade and approved for indefinite implantation, but a strategy should be in place to cover the possible user demand for removal.

### Knowledge gaps and future work

Setting a target plasma level was difficult because most LC_50_ studies employ *in vitro* membrane feeds of a blood-IVM mixture to mosquitoes. This poses a problem because IVM accumulates in adipose tissue [48], which could lead to a higher concentration in skin capillaries than in a vein. In fact, mosquitoes fed on subjects treated with IVM have a three-fold mortality compared to those feeding through a membrane [29]. A threshold of 7 ng/ml was used as target because it is the lowest determined LC_50_ for *An. gambiae* [30]. There is need for a clear determination of the *in vivo* LC_50_ for the main vector species.

Importantly, different vectors have different sensitivity to the drug [49,50]. The LC_50_ varies widely between mosquitoes; *Aedes* have a much higher LC_50_ than *Anopheles* [50] and even different anopheline species in the same area can have different LC_50_ [49]. This should be taken into account when designing slow-release formulations for any specific area or programme.

Using IVM as a transmission-blocking strategy poses many ethical questions. After all, it would mean exposing individuals to the possible side effects of a drug in the name of a community benefit. Many of these questions have been debated regarding transmission-blocking vaccines. The general consensus is that the indirect personal benefit obtained by reducing transmission at the community level would justify the individual use of the drug [51]. Individuals receiving IVM would also benefit from its wide anti-parasitic effects.

The modelling results reveal the exciting potential of a long-lasting IVM product. Even duration as short as two weeks is estimated to reduce the total number of cases of malaria in under fives by 20% in the following six months. In combination with an ACT, a long-lasting IVM drug could play an important role in interrupting malaria transmission by suppressing the infectious vector population in the months following the intervention and preventing resurgence.

## Conclusion

This animal model shows it is technically possible to safely sustain mosquitocidal concentrations of IVM using a slow-release formulation. The total release area and the proportion of the different excipients play an important role in the delivery of the drug. The modelled potential impact on vector population, transmission and clinical incidence is remarkable. These findings warrant further research on slow-release systems for IVM.

## Additional files

Additional file 1:
**Toxicology report.** A complete report of the clinical and analytical toxicological assessment of the rabbits. Additional file 2:
**Vital signs and ECG data.** Table with all the vital sign and ECG measurements done as part of the toxicology workup. Additional file 3:
**Biochemistry data.** Table with all the biochemistry measurements done as part of the toxicology workup.

## Abbreviations

AUC: Area under the curve
DOC: Deoxycholate
EIR: Entomological inoculation rate
GLP: Good laboratory practices
IRS: Indoor-residual spraying
ITNs: Insecticide-treated nets
IVM: Ivermectin
LC_50_: Lethal concentration 50
MDA: Mass drug administration
PK: Pharmacokinetics
SUC: Sucrose
