# Supplementary material for: Screening for an ivermectin slow-release formulation suitable for malaria vector control
Source: Malar J. 2015 Mar 5;14:102. doi: 10.1186/s12936-015-0618-2 (PMC4355127; doi:10.1186/s12936-015-0618-2)
Supplement: Additional file 1: — Toxicology report. A complete report of the clinical and analytical toxicological assessment of the rabbits. [file 12936_2015_618_MOESM1_ESM.pdf]

Screening for an ivermectin slow-release formulation suitable for malaria vector control

Online appendix 1. Toxicology report.

Equipment used

Biochemistry: Roche Hitachi 911 Chemistry Analyzer

Haematology: Sysmex XT1800i hematology analyzer

Coagulation panel: Diagnostica Stago ST4 coagulation analyzer

Urianalysis: Cobas u 411 urine analyzer

Weight

The mean initial weight of the rabbits was 4.3 kg (95%CI 4.2 - 4.4). The mean weight gain was 0.5 kg (95%CI 0.40-0.62) for 12 weeks and 0.97 kg (95%CI 0.85-1.0) for 24 weeks. The mean weight at 12 weeks was 4.8 kg (95%CI 4.6-5.0) and 5.4 kg (95%CI 5.2-5.6) at 24 weeks.

Vital signs

At baseline, heart rate and oxygen saturation were consistent with previously published normal values in rabbits [Manning PJ: The biology of the laboratory rabbit. 2nd edition 2014]. The mean blood pressure has normally been reported in the literature using intravascular measurements; our non-invasive measurements, however, showed a lower mean blood pressure at baseline (55 mmHg, 95% CI 50-60). Additionally, our subjects had a higher than reported mean baseline respiratory rate (69 bpm, 95% CI 65-73) and a lower mean baseline rectal temperature (37.1 ºC, 95% CI 36.7-37.4). We found no significant difference between vital signs at baseline and those at 1, 12 and 24 weeks post-intervention, except a lower mean blood pressure on week 1 (45 mmHg; 95% CI 49-59, $p < 0.05$) and a higher rectal temperature at week 24 (38.4 vs 37.1ºC; $p < 0.01$).

ECG

We found no significant electrocardiographic abnormality. The heart rate and corrected QT interval duration remained within normal range in all groups throughout the study. The mean corrected QT of all subjects at week 24 was 8 milliseconds shorter than at baseline, this difference was statistically significant (p = 0.02) but unlikely to have clinical importance. See additional file 2 for all vital signs and ECG data.

Ophthalmoscopy

We found no signs of toxicity in the indirect ophthalmoscopy in any rabbit at 12 or 24 weeks, regardless of its group.

Hematology and coagulation

Values for red and white blood cell counts, haemoglobin, haematocrit, RBC indices and platelet count remained within normal range in all groups throughout the study.

Baseline coagulation values were normal in all but two animals with prolonged baseline prothrombin time (PT), one animal with a prolonged baseline activated partial thromboplastin time (aPTT), one control with a low fibrinogen and one control with an elevated fibrinogen.

The mean PT of all rabbits 12 weeks post intervention was 0.8 seconds shorter, this difference was statistically significant (95% CI 0.08-1.5 p=0.03). However, the PT values of all subjects receiving IVM remained within the normal range throughout the study. No difference was found at 24 weeks. The aPTT remained within the normal range throughout the study in all but one animal in group 1F with a prolonged aPTT (+15% cut-off value) at 12 weeks.

The mean fibrinogen of all groups was 85 mg/dL higher at 12 weeks independently of the treatment received. This difference was significant (95% CI 48-122 P<0.01). 8 out of 30 animals had abnormally high fibrinogen at 12 weeks (including one control). In the rabbits selected to continue until 24 weeks, 8 out of 14 (including one control) persistently had above normal fibrinogen (p< 0.01 Wilcoxon´s paired sample tests). See table below for all coagulations values.

Group	Baseline			12 weeks			24 weeks		
	Fibrinogen	aPTT	TP	Fibrinogen	aPTT	TP	Fibrinogen	aPTT	TP
1F	360 (209 - 448)	49 (43 - 60)	6.8 (6.7 - 7.1)	417 (387 - 417)	47 (44 - **88**)	6.9 (6.5 - 70)			
2F	364 (364 - 365)	52 (48 - **81**)	6.8 (6.7 - **14.6**)	386 (364 - 400)	47 (47 - 50)	6.5 (6.4 - 6.9)			
3F	349 (340 - 411)	49 (48 - 51)	6.6 (6.3 - 7.0)	**505** (441 - **562**)	43 (42 - 47)	6.4 (6.2 - 6.6)	**472 (468 - 533)**	48 (45 - 51)	7.0 (6.7 - 7.6)
1M	327 (305 - 395)	48 (30 - 55)	6.7 (6.6 - 7.0)	400 (332 - **529**)	46 (42 - 56)	6.7 (6.4 - 7.3)			
2M	322 (272 - 373)	52 (58 - 64)	6.6 (6.5 - 7.1)	**470** (316 - **470**)	58 (44 - 59)	6.7 (6.5 - 75)			
3M	340 (154 - 380)	45 (32 - 48)	6.6 (6.5 - 7.5)	**485** (429 - **487**)	41 (38 - 55)	7.4 (5.7 - 78)	**491** (365 - **505**)	50 (42 - 54)	6.6 (6.1 - 6.7)
1X	133 (133 - 330)	38 (28 - 47)	7.9 (6.8 - **13.1**)	323 (272 - 370)	43 (38 - 48)	6.4 (6.1 - 6.7)			
2X	311 (295 - 344)	50 (50 - 52)	6.6 (6.6 - 6.9)	368 (307 - **463**)	45 (38 - 46)	5.8 (5. - 6.2)	420 (388 - **592**)	52 (44 - 53)	6.7 (6.5 - 7.3)
3X	282 (266 - 345)	51 (31 - 64)	6.9 (6.4 - 7.0)	346 (214 - 438)	37 (31 - 44)	6.2 (6.0 - 6.2)	**570** (358 - **667**)	44 (41 - 47)	6.7 (6.6 - 70)
Controls	235 (**135** - **603**)	54 (36 - 72)	6.8 (6.3 - 7.9)	348 (328 - **586**)	40 (38 - 68)	5.8 (5.6 - 6.3)	439 (439 - **463**)	40 (40 - 59)	6.5 (6.5 - 70)

All values presented as median and range. Fibrinogen (mg/dL), aPTT: activated partial thromboplastin time (seconds), PT: prothrombin time (seconds)

Biochemistry

Values of plasma creatinine, urea, AST, ALT, bilirubin, total proteins, total cholesterol and potassium remained within normal range in all rabbits throughout the study. Our cohort had a plasma sodium value at baseline (160 mEq/L 95%CI 156-163) higher than the normal value reported in the literature (147 mEq/L 95%CI 138-156). The mean plasma sodium (and the 95% CI) remained unchanged regardless of the groups throughout the study. Our cohort had higher than reported blood glucose at baseline (mean 160 mg/dL), mean blood glucose remained unaltered for the whole study. We observed a significantly higher albumin in rabbits kept until 24 weeks. See additional file 3 for all biochemistry values. No significant alteration was found in the urinalysis 24 hours before sacrifice.

Clinical events

Three clinically relevant incidents were reported: One subject in group 1X developed an abscess in the anterior neck skin (20 cm away from the insertion site) seven days after implantation; he received treatment with NSAIDs and oral enrofloxacin for 2 weeks and recovered fully. One rabbit in group 1M presented self-limited hyporexia and weight loss 3 weeks after implantation, veterinary and laboratory assessment showed no anomaly, recovery occurred in less than 2 weeks. One rabbit in group 2M presented unilateral testicular inflammation 85 days after implantation, he received treatment with NSAIDs and oral enrofloxacin.

Deaths

Three rabbits died during the study. One rabbit in group 2X was sacrificed for ethical reasons 6 days after implantation when he developed an abscess in the abdominal skin (30 cm away from the insertion site). One rabbit in group 3M died of postural apnoea in the transport box while recovering from the anaesthesia for the ECG 7 days after implantation and one rabbit in group 1M was sacrificed for ethical reasons 8 days after implantation, he lost sensitive and motor functions in the legs after a prolonged apnoea during blood sampling under sedation.

Autopsies

During autopsies no macroscopic abnormalities in liver, heart, kidneys, adrenal glands and testicles were noticed. As expected, the organs of the subjects sacrificed at 24 weeks were proportionally smaller since weight in adulthood is disproportionally gained in adipose tissue. It was of notice, however, that the proportional spleen weight at 12 weeks was not larger in the controls. For this reason, although no significant size differences were found in the thymus, we decided to submit spleen and thymus for histological examination. No microscopic abnormalities were found.
